# Pictorial essay: Susceptibility-weighted imaging in cerebral ischemia

**DOI:** 10.4103/0971-3026.73530

**Published:** 2010-11

**Authors:** Puneet Mittal, Vishal Kalia, Sarika Dua

**Affiliations:** Department of Radiodiagnosis, Punjab Institute of Medical Sciences, Jalandhar, Punjab, India; 1 Department of Radiodiagnosis, Dayanand Medical College & Hospital, Ludhiana, India

**Keywords:** Ischemia, magnetic, susceptibility

## Abstract

The susceptiblity effect in magnetic resonance imaging (MRI) has been recognized for long and often has been considered undesirable, producing unnecessary noise. Susceptibility-weighted imaging (SWI) aims at exploiting this effect to provide a different type of contrast that is suited for vascular imaging. We describe five different cases in which SWI was found useful to delineate the underlying ischemia or to arrive at the corect diagnosis.

## Introduction

Susceptibility-weighted imaging (SWI) is a new technique that exploits susceptibility differences in different tissues, to provide a different type of tissue contrast.[1] It is particularly suited for vascular imaging, especially in cerebral ischemia.[2] It is exquisitely sensitive to blood products, even more than the gradient-echo (GRE) technique.[3,4] It also provides a unique tissue contrast, similar to blood oxygen level–dependent (BOLD) imaging.[4,5] It can provide important diagnostic information and also provide insights into etiolopathogenesis.[1]

## Materials and Methods

We present a series of five cases, wherein we compared SWI with other imaging sequences and found it to be useful for reaching a diagnosis, confirming it, and providing insights into the etiopathogenesis.

In all cases described later in the text, SWI was performed on an 18-channel, 1.5 Tesla scanner (Avanto, Siemens, Erlangen, Germany) using the following parameters: time to repetition (TR) — 50 ms, time to echo (TE) — 40 ms, flip angle — 20°, slice thickness — 3 mm, bandwidth — 80 kHz, field of view (FOV) read — 230 mm, FOV phase — 87.5%, base resolution — 256, phase resolution — 75%, and integrated parallel acquisition technique (IPAT) factor — 2. Separate phase, magnitude, SWI, and minimum intensity projections were generated and analyzed.

### Case 1

**Figure 1 (A–D):**
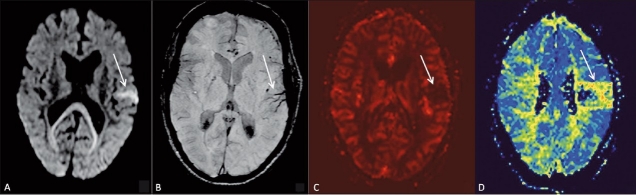
A 46-year-old male with acute onset of right hemiparesis. Diffusion-weighted image (A) shows an area of restricted diffusion (arrow) in the left temporal lobe. SWI (B), cerebral blood volume (CBV) map (C), and mean transit time (MTT) map (D) show matching areas of abnormality (arrows). No mismatch is seen

A 46-year-old male patient presented with acute onset of right hemiparesis for two hours prior to admission. Diffusion-weighted images showed a small area of diffusion restriction in the left temporal region [Figure 1A]. On SWI [Figure 1B], prominent hypointense signal was seen in the vessels in the same region. On MR perfusion scan, cerebral blood flow (CBF) [Figure 1C] and mean transit time (MTT) [Figure 1D] maps showed a similar, matching, defect. A follow-up scan did not show any progression of the infarct size.

### Case 2

**Figure 2 (A–D):**
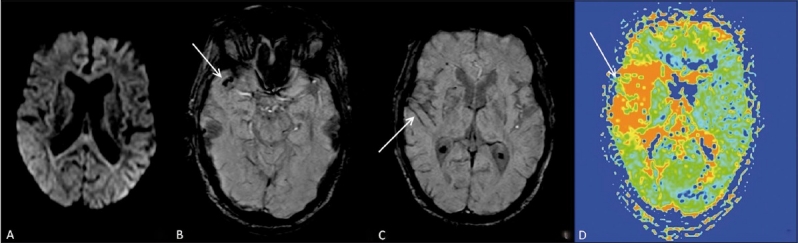
A 52-year-old male with transient weakness of the left side of the body. Axial diffusion-weighted image (A) shows no abnormality. SWI image (B) shows a thrombus (arrow) at the right MCA bifurcation (the susceptibility sign). SWI image at a higher level (C) shows prominent cortical vessels (arrow) on the right side. Perfusion mean transit time (MTT) map (D) confirms a focal perfusion abnormality (arrow) in the same region

A 52-year-old male patient presented with transient weakness of the left side of the body. Routine sequences, including diffusion-weighted images [Figure 2A] did not reveal any abnormality. On SWI images [Figure 2B], a hypointense signal with blooming was seen at the distal right middle cerebral artery (MCA) bifurcation, indicating thrombosis (the susceptibility sign). A few prominent hypointense vessels were seen along the left cerebral hemisphere, indicating ischemia [Figure 2C]. On an MTT map [Figure 2D], a perfusion defect was seen in the left MCA territory.

### Case 3

**Figure 3 (A–C):**
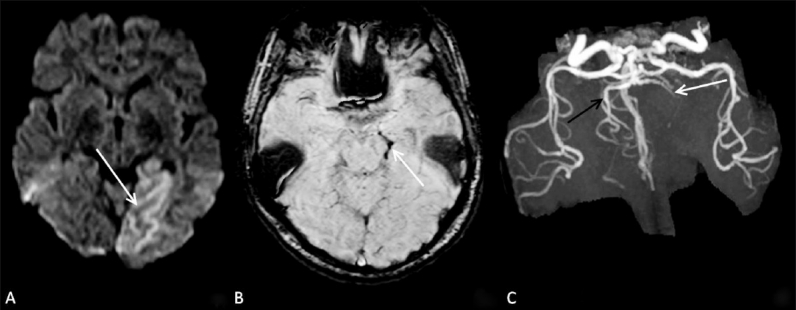
A 56-year-old female with acute stroke in the left PCA territory. Diffusion-weighted image (A) shows an acute infarct (arrow) in the left PCA territory. SWI image (B) shows blooming (arrow) in the P2 segment of the left PCA. TOF MRA (C) confirms thrombus (arrow) in the left PCA.

A 56–year-old female presented with acute onset of altered sensorium, headache, and aphasia. She was suspected to have a posterior circulation stroke. On diffusion-weighted images [Figure 3A], an acute infarct was seen in the left posterior cerebral artery (PCA) territory. On SWI [Figure 3B], a hypointense signal with blooming was seen in the P2 segment of the left PCA. On time-of-flight MRI angiography (TOF MRA) [Figure 3C], flow-related enhancement was not seen in the left PCA, thus confirming the findings on SWI.

### Case 4

**Figure 4 (A–D):**
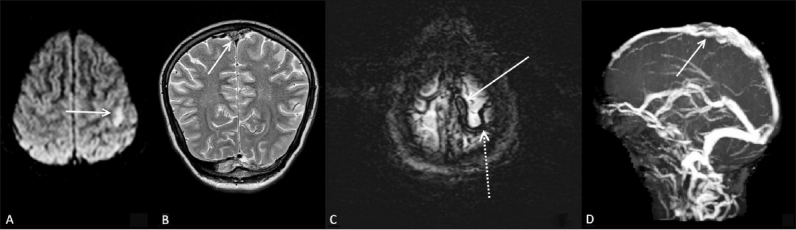
A 27-year-old male with weakness and paresthesia in the right upper limb. Diffusion-weighted image (A) shows a focal hyperintensity (arrow) in the left parietal region, representing an acute infarct. Coronal T2W image (B) shows intermediate signal (arrow) in the superior sagittal sinus, representing an acute thrombus. Axial SWI image (C) shows thrombosis in the superior sagittal sinus (arrow) and an adjacent cortical draining vein (dotted arrow) on the left side. TOF MRI venography (D) confirms expansion and thrombosis (arrow) of the superior sagittal sinus

A 27-year-old male presented with weakness and paresthesia in the right upper limb for one day prior to presentation. Diffusion-weighted images [Figure 4A] showed a small infarct in the left parietal region. Coronal T2W images [Figure 4B] showed intermediate signal intensity in the high superior sagittal sinus. SWI images [Figure 4C] showed blooming and thrombus in the superior sagittal sinus and a cortical vein draining into it. Venous thrombosis was confirmed on TOF MRI venography [Figure 4D].

### Case 5

**Figure 5 (A–D):**
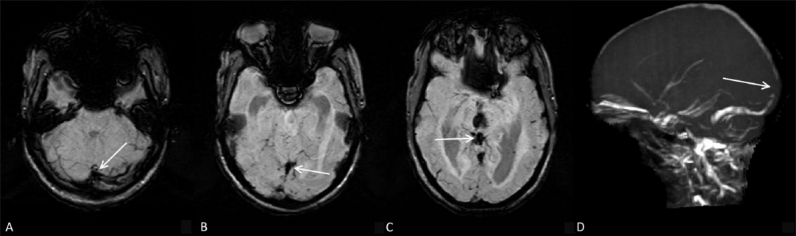
A 30-year-old female with acute onset of headache and altered sensorium. Axial SWI images (A–C) show thrombosis (arrows) at the level of the torcula (A), the straight sinus (B), and the vein of Galen (C), which is confirmed on TOF MRI venography (arrow in D)

A 30-year-old female presented with sudden onset of headache and altered sensorium. SWI images showed thrombosis at the torcula and in the deep venous system [Figure 5A and B]. MRI venography [Figure 5C] confirmed the diagnosis of deep cerebral venous thrombosis.

## Discussion

Susceptibility-weighted imaging (SWI) is a relatively new and unique sequence. Although in most of the traditional MRI techniques there is a constant effort at decreasing the susceptibility effects that produce noise, SWI aims at exploiting the same. It is a 3D sequence that exploits the differences in the susceptibility properties of different tissues.[1] Therefore, it provides a unique contrast that is not afforded by conventional sequences. It is extremely sensitive to even minute amounts of paramagnetic substances.[1,2,4] In this respect it is even more sensitive than the gradient-echo (GRE) sequences, partly because of its inherent sensitivity, increased spatial resolution and the thinner slices acquired.[2–4]

Susceptibility-weighted imaging provides a unique contrast, similar to blood oxygen level–dependent (BOLD) imaging, which is widely used in functional imaging.[3] When there is hypoperfusion of any region of the brain due to deficient arterial supply, it promotes focal vasodilatation. This causes relative slowing of the circulation and increased extraction of oxygen from the blood in the ischemic region. This causes a focal increase in the concentration of deoxyhemoglobin in the venous blood. As deoxyhemoglobin is paramagnetic, this can be detected by SWI. Therefore, venous vessels in the ischemic region appear hypointense and prominent.[2,3,5] This focal concentration of hypointense vessels can be potentially used as a marker for ischemic regions in the brain. The same principle can be used for identifying the ischemic penumbra (at-risk tissue) in acute stroke. Thus, a diffusion — susceptibility mismatch could potentially provide information similar to that obtained from a diffusion — perfusion mismatch.[3] In case 1, we see that the affected area on diffusion, SWI, and perfusion images are almost similar. On repeat diffusion imaging 24 hours later, no extension of the infarct was seen. A diffusion-perfusion mismatch is not as accurate in predicting the ischemic penumbra as was originally thought.[6] It has been suggested that BOLD-related MRI imaging may provide a better estimation of the potentially salvageable zone.[7] SWI-diffusion mismatch may provide similar information, however, further studies are required to evaluate its usefulness.

Acute thrombus contains deoxyhemoglobin. Therefore, acute arterial thrombosis can be identified on SWI images by what is known as the susceptibility sign.[3,4] This comprises of a focal hypointense signal in the vessel and apparent enlargement of its diameter, due to blooming, related to its paramagnetic properties. The susceptibility sign is seen in cases 2 and 3. Case 2 also demonstrates the ability of SWI to detect ischemia even when diffusion-weighted images are normal, as was eventually confirmed by perfusion imaging in our case. This can alert the clinician to the potential for subsequent infarction and leads to appropriate preventive measures.

Susceptibility-weighted imaging is also a good technique to demonstrate venous sinus thrombosis, as is demonstrated in Cases 4 and 5. Thrombosed sinuses have deoxyhemoglobin, which can be readily detected on SWI by the presence of hypointensity and blooming.[5,8] Furthermore, venous infarcts are frequently hemorrhagic and SWI assists in detecting even small hemorrhages in venous infarcts.

In conclusion, SWI is a relatively new technique that provides a unique contrast and new possibilities, especially in the imaging of vascular and ischemic conditions. Further studies are needed for delineating its current role in imaging, but it certainly adds significantly to the armamentarium of the sequences available.
